# Antifibrotic effects of 2-carba cyclic phosphatidic acid (2ccPA) in systemic sclerosis: contribution to the novel treatment

**DOI:** 10.1186/s13075-019-1881-3

**Published:** 2019-04-18

**Authors:** Tomoaki Higuchi, Kae Takagi, Akiko Tochimoto, Yuki Ichimura, Takanari Norose, Yasuhiro Katsumata, Ikuko Masuda, Hisashi Yamanaka, Toshiro Morohoshi, Yasushi Kawaguchi

**Affiliations:** 1 Department of Rheumatology, Tokyo Women’s Medical University School of Medicine, 8-1, Kawada-cho, Shinjuku-ku, Tokyo, 162-8666 Japan; 2 SANSHO, Co., Ltd., Tokyo, Japan

**Keywords:** Fibrosis, Fibroblasts, Systemic sclerosis, Cyclic phosphatidic acid, Treatment

## Abstract

**Background:**

Cyclic phosphatidic acid (cPA) has an inhibitory effect on the autotaxin (ATX)/lysophosphatidic acid (LPA) axis, which has been implicated to play an important role in the progression of fibrosis in systemic sclerosis (SSc). The purpose of this study is to assess the antifibrotic activity of cPA for the treatment of SSc using SSc skin fibroblasts and an animal model of bleomycin-induced skin fibrosis.

**Methods:**

We used a chemically stable derivative of cPA (2ccPA). First, we investigated the effect of 2ccPA on extracellular matrix (ECM) expression in skin fibroblasts. Next, the effect of 2ccPA on the intracellular cAMP levels was determined to investigate the mechanisms of the antifibrotic activity of 2ccPA. Finally, we administered 2ccPA to bleomycin-induced SSc model mice to evaluate whether 2ccPA prevented the progression of skin fibrosis.

**Results:**

2ccPA decreased ECM expression in SSc skin fibroblasts and TGF-β1-treated healthy skin fibroblasts without LPA stimulation. 2ccPA increased the intracellular cAMP levels in skin fibroblasts, suggesting that the antifibrotic effect of 2ccPA was the consequence of the increase in the intracellular cAMP levels. Administration of 2ccPA also ameliorated the progression of bleomycin-induced skin fibrosis in mice.

**Conclusions:**

Our data indicated that 2ccPA had inhibitory effects on the progression of skin fibrosis by abrogating ECM production from activated skin fibroblasts. These cells were repressed, at least in part, by increased intracellular cAMP levels. 2ccPA may be able to be used to treat fibrotic lesions in SSc.

## Background

Systemic sclerosis (SSc) is a systemic connective tissue disease with excessive fibrosis and vascular malformation based on autoimmunity [1–3]. Progressive fibrosis, which affects vital organs, such as the lungs and the gastrointestinal tract, is sometimes fatal or severely impairs quality of life [4]. Fibrosis of the skin, namely, scleroderma, is a major therapeutic target of SSc. Several molecules, such as transforming growth factor-β (TGF-β) and platelet-derived growth factor (PDGF), have been reported to be closely associated with the progression of fibrosis [1–3, 5–7]. However, treatments including blocking agents of these molecules have not previously been established because of their limited therapeutic effects and/or severe adverse events [5–7].

An autotaxin (ATX)/lysophosphatidic acid (LPA) axis has emerged as a novel pathogenic factor in various diseases, including fibrosing disorder [8–10]. ATX is a secreted form of lysophospholipase D. One of the major properties of ATX is LPA production via cleavage of the choline group from lysophosphatidylcholine (LPC) [10]. LPA binds to six specific G protein-coupled receptors (GPCRs) (LPA_1–6_) and intracellular PPARγ [11, 12]. LPA is known to regulate several cellular properties, including survival, proliferation, differentiation, and motility [8–10]. These physiological features have been implicated in the pathogenesis of conditions such as autoimmunity, fibrosis, cancer, and inflammation; thus, LPA has been identified as a therapeutic target in various intractable diseases [11, 12]. Among LPA receptors, the LPA_1_-dependent cellular pathway has been particularly well studied for the treatment of fibrosis and LPA_1_ antagonists have entered phase II clinical trials for the treatment of idiopathic pulmonary fibrosis and other fibrogenic conditions [13]. The serum levels of 2-arachidonoyl-LPA (also referred to as S1P) in the sera of SSc patients were elevated, and skin fibroblasts obtained from affected areas of SSc patients had more Cl^−^ current activated by LPA and S1P, which play critical roles in myofibroblast differentiation [14, 15]. In animal models, LPA_1_ knockout mice showed less extensive skin fibrosis in bleomycin-induced skin fibrosis [16].

Cyclic phosphatidic acid (cPA) is a naturally occurring lipid mediator that was first isolated from a slime mold [17]. cPA also exists in human sera. Similar to LPA, cPA is catalyzed by ATX; however, cPA has several distinct activities from LPA due to the presence of cyclic phosphates at the sn-2 and sn-3 portions of its glycerol carbons [17]. Moreover, cPA has been reported to have an antagonizing effect on the ATX/LPA axis. Although cPA is rapidly catalyzed, the 2-carba derivative of cPA (2ccPA) is stable in humans and is expected to be used in various clinical settings [17–19]. From these findings, we hypothesized that cPA has antifibrotic effects on SSc. Herein, we found that 2ccPA inhibited the production of type I collagen and other fibrotic molecules in SSc skin fibroblasts and TGF-β-treated healthy skin fibroblasts. Furthermore, 2ccPA also prevented the progression of bleomycin-induced skin fibrosis in mice. These findings suggest that 2ccPA may be effective for the treatment of fibrotic lesions of the skin and other internal organs in SSc.

## Methods

### Skin fibroblasts

SSc skin fibroblasts were obtained from lesional areas of the forearms of SSc patients. The diagnosis of SSc was based on the 2013 ACR/EULAR classification criteria for SSc [20]. All patients were diffuse cutaneous type positive for anti-topoisomerase I antibodies and had never received an immunosuppressive treatment. This study was approved by the ethics committee of Tokyo Women’s Medical University. We informed all participants of the contents of this study, and written consent was obtained. Healthy adult skin fibroblasts were purchased (Lonza, Basel, Switzerland; Kurabo, Osaka, Japan). Skin fibroblasts used in the experiment were up to five passages. Skin fibroblasts were incubated with Dulbecco’s modified Eagle’s medium (Hyclone, Logan, UT, USA) supplemented with 10% fetal calf serum (Sigma-Aldrich, St. Louis, MO, USA) at 37 °C as previously described [21]. Subconfluent fibroblasts were cultured in serum-free DMEM for 24 h. After serum starvation, fibroblasts were incubated with various concentrations of 2ccPA (SANSHO, Tokyo, Japan) and forskolin (MP Biomedicals, Santa Ana, CA, USA) in the presence or absence of LPA (Sigma-Aldrich) or TGF-β1 (R&D Systems, Minneapolis, MN, USA).

### Cell viability

Cell viability was calculated using a Cell Counting Kit 8 (Dojindo, Kumamoto, Japan) according to the manufacturer’s instructions.

### Quantitative real-time reverse transcription-polymerase chain reaction

Total RNA contained in skin fibroblasts was collected using a commercially available kit (Life Technologies, Carlsbad, CA, USA). Equal amounts of total RNA were reverse-transcribed to synthesize cDNA using a SuperScript VILO cDNA Synthesis kit (Thermo Fisher Scientific, Waltham, MA, USA) according to the manufacturer’s instructions. TaqMan primers (Thermo Fisher Scientific) were used in our experiments. cDNA was mixed with a master mix (Thermo Fisher Scientific), and each primer was then applied to a plate in triplicate. DNA was amplified on a ViiA 7 Real-time PCR system (Thermo Fisher Scientific). The TaqMan primer and probe sets used in our experiment were as follows: Hs00164004_m1 (*COL1A1*), Hs00164099_m1 (*COL1A*2), Hs00171257_g1 (*CTGF*), Hs00426835_g1 (*ACTA2*), Hs00365052_m1 (*FN*), Hs00171257_m1 (*TGF-β1*), and Hs00174131_m1 (*IL-6*). *GAPDH* was used as the endogenous control, and the expression level of each mRNA was calculated using the delta-delta CT method. We performed at least three independent experiments for qPCR analysis.

### Western blotting

Cultured skin fibroblasts were lysed with lysis buffer. The concentration of proteins in the lysis buffer was calculated using the BCA assay. Equal amounts of protein were applied in Tris-glycine gel (Thermo Fisher Scientific), and proteins were separated by SDS-PAGE. Gels were transferred onto polyvinylidene fluoride (PVDF) membranes, and the membranes were then blocked with 5% nonfat milk in TBS-T for 1 h at room temperature. The membranes were incubated with primary antibodies overnight at 4 °C. The primary antibodies were as follows: unlabeled goat anti-type I collagen antibodies (1310-01) (1:1000, Southern Biotechnology, Birmingham, AL, USA), polyclonal goat anti-CTGF antibodies (sc-14939) (1:1000; Santa Cruz Biotechnology, Santa Cruz, CA, USA) [22], rabbit anti-αSMA antibodies (14968 s) (1:250; Cell Signaling Technologies, Denver, MA, USA), and polyclonal rabbit anti-GAPDH antibodies (sc-25778) (1:1000; Santa Cruz Biotechnology). After washing with TBS-T three times, the membranes were incubated with polyclonal rabbit anti-goat (MBL 546) and polyclonal goat anti-rabbit (MBL 458) secondary antibodies (1:500,000; Medical & Biological Laboratories, Nagoya, Aichi, Japan). The bands were visualized using an ECL solution (Wako, Osaka, Japan). The density of the bands was calculated using ImageJ software (NIH, Bethesda, MD, USA).

### Cyclic AMP (cAMP) measurement

Fifteen minutes prior to cell lysate collection, cells were treated with 3-isobutyl-1-methylxanthine (IBMX) to eliminate the effects of endogenous PDE activities. The intracellular cAMP levels were then assessed using an enzyme immunoassay kit (Cayman Chemical, Ann Arbor, MI, USA) according to the manufacturer’s instructions.

### Procollagen type I measurement

The levels of procollagen type I were measured using a commercially available enzyme immunoassay kit (Takara Bio, Kusatsu, Shiga, Japan) according to the manufacturer’s instructions.

### Mice

Six-week-old female BALB/c mice (Sankyo Labo Service, Tokyo, Japan) were used in our experiment [23]. To develop bleomycin-induced skin fibrosis, mice were shaved on their backs and subcutaneously injected with 300 μg of bleomycin (1 mg/ml dissolved in PBS) (Nihon Kayaku, Tokyo, Japan) five times per week for 4 weeks as previously described [24, 25]. An equal amount of PBS was subcutaneously injected into control mice. In 2ccPA-treated mice, the indicated amounts of 2ccPA (dissolved in PBS at a concentration of 1 mg/ml or 100 μg/ml) were intraperitoneally administered concurrently with bleomycin to assess the preventive effect on skin fibrosis. In control mice, an equal amount of PBS was intraperitoneally injected. After completing the protocol, the back skin was removed. The skin was fixed in 10% formaldehyde and embedded in paraffin. All experimental protocols were approved by the Ethical Review Committee of Animal Experiments, Tokyo Women’s Medical University.

### Assessment of dermal thickness

The slides were stained with Masson’s trichrome staining. The distance between the epidermal-dermal junction to the dermal-fat junction was measured for the assessment of the dermal thickness. The average of the dermal thickness of five randomly selected different fields at an equal magnification (× 100) was calculated according to a previous study [26].

### Immunohistochemistry

The sections were deparaffinized and incubated with citrate buffer (pH 9.0) at 95 °C for 20 min, and the sections were then incubated with 3% H_2_O_2_ and blocked with 5% nonfat milk in PBS. The samples were reacted with polyclonal rabbit anti-αSMA antibodies (ab5694) (1:2000; Abcam, Cambridge, UK) at 4% overnight. Antibodies were visualized using an anti-rabbit DAKO EnVision horseradish peroxidase system (DAKO, Carpinteria, CA, USA). The sections were developed with 3,3′-diaminobenzidine tetrahydrochloride dihydrate and counterstained with hematoxylin. Finally, we counted the average number of αSMA-positive cells in the dermis of five randomly selected different fields at an equal magnification (× 100) according to previous reports [27].

### Collagen content

Equal areas of skin were collected from the mice using a 6-mm-diameter biopsy punch (KAI industries, Seki, Gifu, Japan). Skin samples were quantified using a QuickZyme total collagen assay (QuickZyme Biosciences, Leiden, Netherlands) according to the manufacturer’s instructions.

### Statistical analysis

Data were analyzed using JMP and R version 3.5.2 [28]. Statistical analyses were performed using one-way ANOVA followed by Student’s *t* test for normally distributed data or the Mann-Whitney *U* test for non-normally distributed data. *p* value < 0.05 was considered statistically significant.

## Results

### 2ccPA suppressed the production of extracellular matrix molecules in healthy skin fibroblasts

**Fig. 1:**
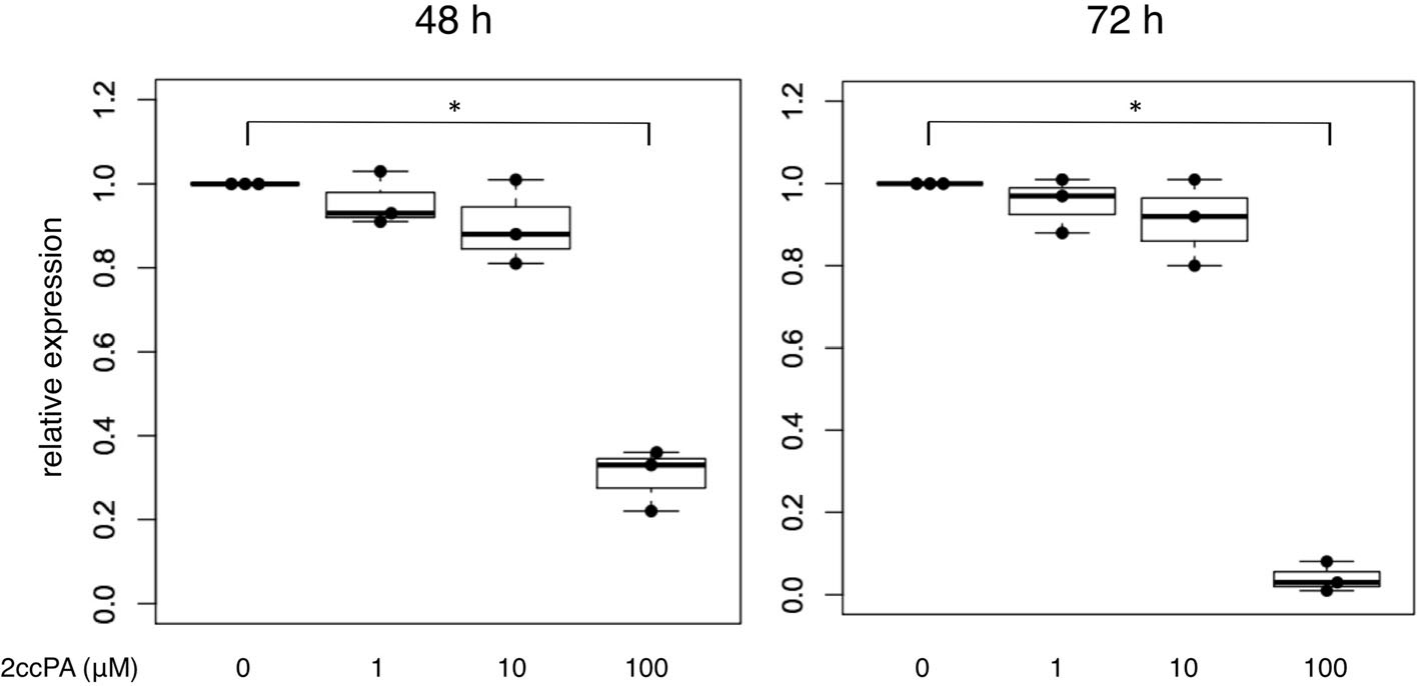
Viability of skin fibroblasts in various concentrations of 2ccPA. Skin fibroblasts derived from three different SSc patients were incubated with 1–100 μM 2ccPA for 48 and 72 h. Phosphate-buffered saline (PBS) was added in the 0 μM 2ccPA group. Cell viability was measured using a commercially available cell proliferation measurement kit (Dojindo, Japan). The bars represent as median with interquartile range (IQR). n.s., not significant; *p < 0.05

First, we tested the viability of 2ccPA in SSc skin fibroblasts. Overall, 1–10 μM 2ccPA did not affect the viability of SSc skin fibroblasts at 48 and 72 h after 2ccPA administration (Fig. 1); a similar result was obtained in normal skin fibroblasts (data not shown).

**Fig. 2:**
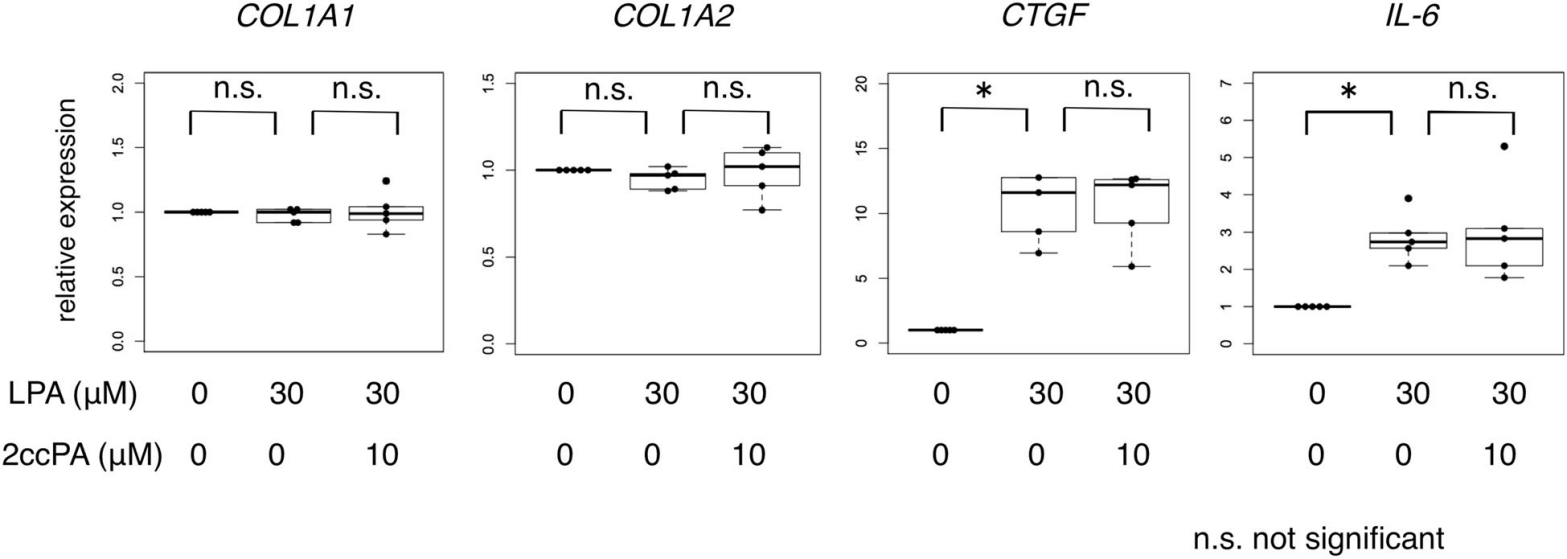
2ccPA did not interfere with the upregulation of LPA-driven profibrotic markers in SSc skin fibroblasts. SSc skin fibroblasts (*n* = 5) were incubated with 30 μM LPA in the presence or absence of 10 μM 2ccPA for 6 h. The expression of *COL1A1*, *COL1A2*, *CTGF*, and *IL-6* mRNA were determined by qPCR. Data are the representative of three independent experiments. The bars represent as median with IQR. *p < 0.05

**Fig. 3:**
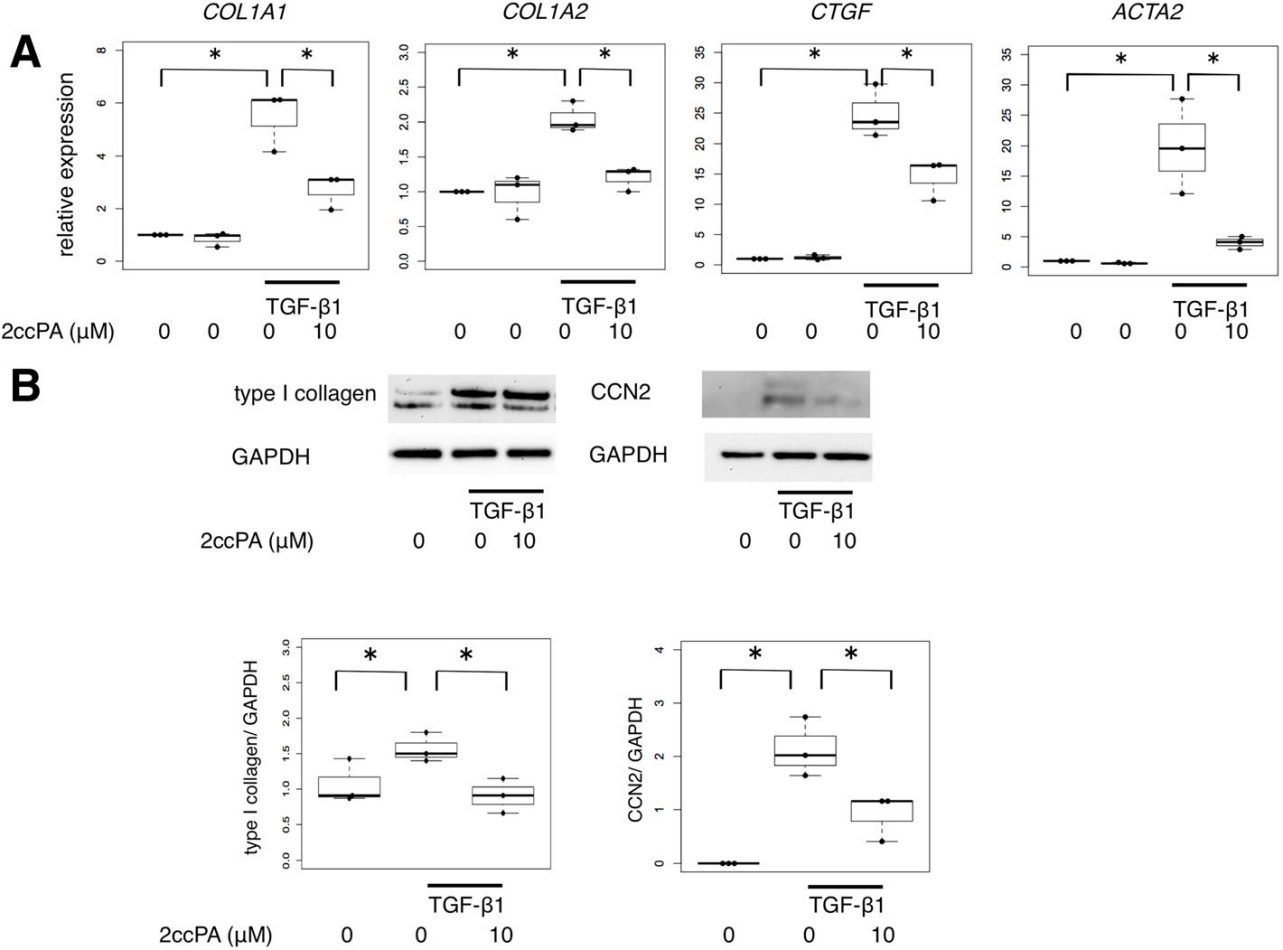
2ccPA inhibited the upregulation of profibrotic markers in normal skin fibroblasts stimulated with TGF-β1. Normal skin fibroblasts (*n* = 3) were incubated with or without 10 μM 2ccPA in the presence or absence of 10 ng/mL TGF-β1 for 48 h. **a** The expression of *COL1A1*, *COL1A2*, *CTGF*, and *ACTA2* mRNA were determined by qPCR. **b** Normal skin fibroblasts (*n* = 3) were incubated with 10 μM 2ccPA in the presence or absence of 10 ng/mL TGF-β1 for 72 h. The protein expression of type I collagen and CCN2 in cell lysates of normal skin fibroblasts were assessed with Western blotting. Data are the representative of three independent experiments. The bars represent as median with IQR. *p < 0.05

The ATX/LPA/IL-6 autocrine loop has been reported to be a potential driver of the fibrogenic phenotype in SSc [29]. First, we determined the fibrogenic potential of LPA in skin fibroblasts. In line with a previous report, LPA treatment increased the mRNA expression levels of *CTGF* and *IL-6* in the healthy and SSc skin fibroblasts at 6 h after LPA treatment (Fig. 2), whereas the *COL1A1* and *COL1A2* mRNA expression levels were not affected by LPA up to 48 h (data not shown). We subsequently assessed our hypothesis that 2ccPA might interfere with the ATX/LPA autocrine loop. However, *CTGF* and *IL-6* mRNA expression in LPA-driven fibrogenic skin fibroblasts was not downregulated by 2ccPA treatment (Fig. 2). We then tested the inhibitory effect of 2ccPA on extracellular matrix (ECM) production in other fibrotic situations. To investigate the effect of 2ccPA on activated skin fibroblasts in response to TGF-β1, healthy skin fibroblasts were treated with 2ccPA in the presence or absence of 10 ng/mL TGF-β1. Similarly, 2ccPA downregulated the mRNA expression levels of ECM molecules, including *COL1A1*, *COL1A2*, *CTGF*, and *ACTA2*, in healthy skin fibroblasts incubated with TGF-β1 (Fig. 3a). To investigate the antifibrotic properties of 2ccPA at the protein level, we performed Western blot analysis. As expected, 2ccPA decreased the protein expression levels of type I collagen, CCN2 and αSMA in healthy skin fibroblasts treated with TGF-β1 (Fig. 3b). Collectively, 2ccPA demonstrated an inhibitory effect of ECM production in skin fibroblasts, regardless of the ATX/LPA axis.

### 2ccPA ameliorated the fibrotic phenotype of SSc skin fibroblasts

**Fig. 4:**
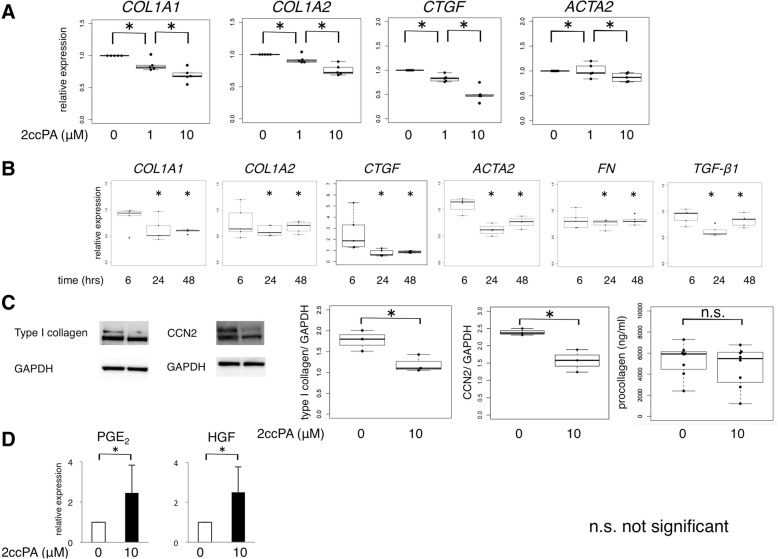
The antifibrotic effect of 2ccPA on SSc skin fibroblasts. **a** SSc skin fibroblasts (*n* = 5) were incubated with 0–10 μM 2ccPA for 48 h. The expression of *COL1A1*, *COL1A2*, *CTGF*, and *ACTA2* mRNA was calculated by qPCR. **b** Time course expression of profibrotic markers in SSc skin fibroblasts (*n* = 5) treated with 2ccPA. SSc skin fibroblasts were incubated with 10 μM 2ccPA for 6–48 h. The expression of *COL1A1*, *COL1A2*, *ACTA2*, *fibronectin* (*FN*), and *TGF-β1* mRNA was calculated by qPCR, and the amount of expression was compared with that of no treatment group. **c** The expression of type I collagen and CCN2 in cell lysates of SSc skin fibroblasts (*n* = 3) treated with 2ccPA. SSc skin fibroblasts were incubated with 0–10 μM 2ccPA for 48 h. The protein expression of cell lysates was detected by Western blotting. To calculate the levels of procollagen type I in the medium, SSc skin fibroblasts (*n* = 8) were incubated with 0–10 μM 2ccPA for 72 h and cultured medium were assessed using a commercially available enzyme immunoassay kit. **d** SSc skin fibroblasts (*n* = 8) were incubated with 10 μM 2ccPA for 24 h. PGE_2_ and HGF levels in the cultured medium were calculated by commercially available enzyme-linked immunosorbent assay kits. The bars represent as median with IQR in **a**–**c**. Data are the representative of three independent experiments in **a**–**c**. Bars represent the mean ± standard deviation (SD) in **d**. *p < 0.05

To further investigate the inhibitory effect of ECM production on SSc skin fibroblasts, we cultured SSc skin fibroblasts with 2ccPA. Consistent with the results in healthy skin fibroblasts, 2ccPA downregulated the expression levels of profibrotic mRNA in SSc skin fibroblasts in a dose-dependent manner (Fig. 4a). Furthermore, we assessed the time course of mRNA expression in response to the 2ccPA treatment. The antifibrotic effect of 2ccPA on SSc skin fibroblasts assessed by qPCR was detectable up to 48 h after treatment (Fig. 4b). Similarly, Western blot analysis indicated that 2ccPA decreased the protein expression levels of ECM components in SSc skin fibroblasts (Fig. 4c). The procollagen levels in the supernatant of 2ccPA-treated SSc skin fibroblasts were not significantly lower than that of the no treatment group (Fig. 4c). Furthermore, we speculated that 2ccPA may stimulate the production of antifibrotic agents in skin fibroblasts. Hepatocyte growth factor (HGF) and prostaglandin E_2_ (PGE_2_) have been reported to have an antifibrotic potential via the inhibition of myofibroblast differentiation [30–32]. Therefore, we calculated the HGF and PGE_2_ levels in a cultured medium in SSc skin fibroblasts. Consistent with our hypothesis, the HGF and PGE_2_ levels were significantly higher in the 2ccPA-treated group than in the untreated group (Fig. 4d). Taken together, 2ccPA could diminish the fibrotic activity of SSc-derived fibroblasts.

### The antifibrotic activity of 2ccPA was partly dependent on the intracellular cAMP levels

**Fig. 5:**
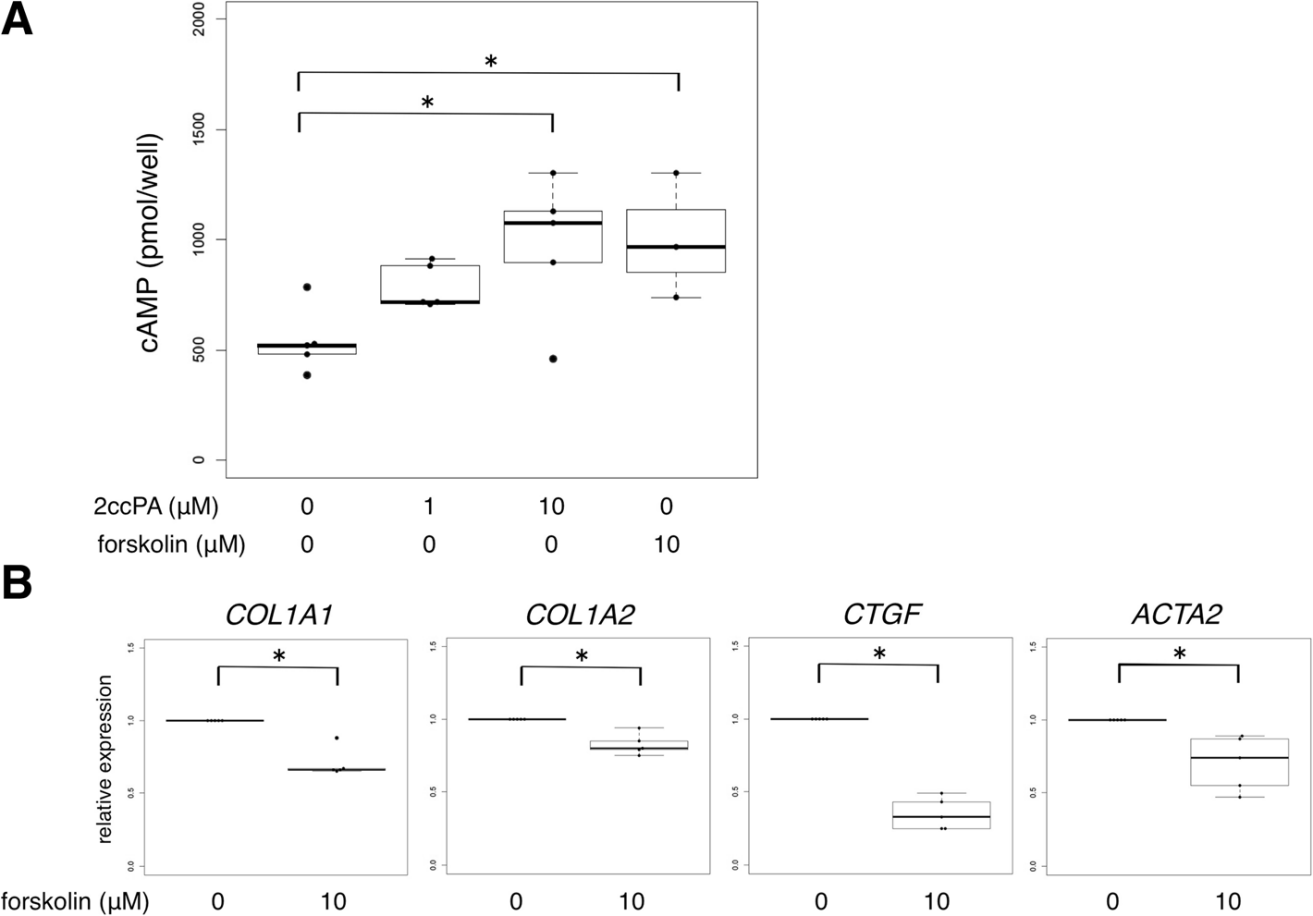
2ccPA regulated the fibrogenic environment in part by increasing intracellular cAMP levels in SSc skin fibroblasts. **a** SSc skin fibroblasts (*n* = 5) were incubated with either 0–10 μM 2ccPA for 30 min in the presence of 3-isobuthyl-1-methylxanthine. One micromolar of forskolin was administrated in SSc skin fibroblasts (*n* = 3) as a positive control. The intracellular cAMP levels were assessed using an enzyme immunoassay kit. **b** Profibrotic markers were downregulated by increasing intracellular cAMP levels in SSc skin fibroblasts. SSc skin fibroblasts (*n* = 5) were incubated in the presence of 0–10 μM forskolin for 48 h. *COL1A1*, *COL1A2*, *CTGF*, and *ACTA2* mRNA was assessed by qPCR. Data are the representative of three independent experiments. The bars represent as median with IQR. *p < 0.05

Both 2ccPA and PGE_2_ have been shown to increase the intracellular cAMP levels in fibrogenic cells, such as mesangial cells in the kidney and copper cells in the liver, along with skin fibroblasts [33]. Furthermore, increased intracellular cAMP levels have been reported to regulate the fibrogenic environment in tissues. Thus, we hypothesized that 2ccPA prevented the production of ECM molecules in skin fibroblasts by regulating intracellular cAMP levels. To confirm this hypothesis, we analyzed the intracellular cAMP levels of SSc skin fibroblasts using a commercially available kit. In line with our hypothesis, 2ccPA significantly increased the intracellular cAMP levels in a dose-dependent manner (Fig. 5a). Furthermore, to test whether the increased intracellular cAMP levels could alter the fibrogenic phenotype of SSc skin fibroblasts, we incubated SSc skin fibroblasts with or without 10 μM forskolin, a direct stimulator of adenylate cyclase (AC). As expected, forskolin decreased the mRNA expression levels of *COL1A1*, *COL1A2*, *CTGF*, and *ACTA2* in SSc skin fibroblasts (Fig. 5b). Collectively, 2ccPA diminished the production of ECM components in SSc skin fibroblasts, at least in part, by increasing the intracellular cAMP levels.

### 2ccPA prevented the progression of bleomycin-induced skin fibrosis in mice

**Fig. 6:**
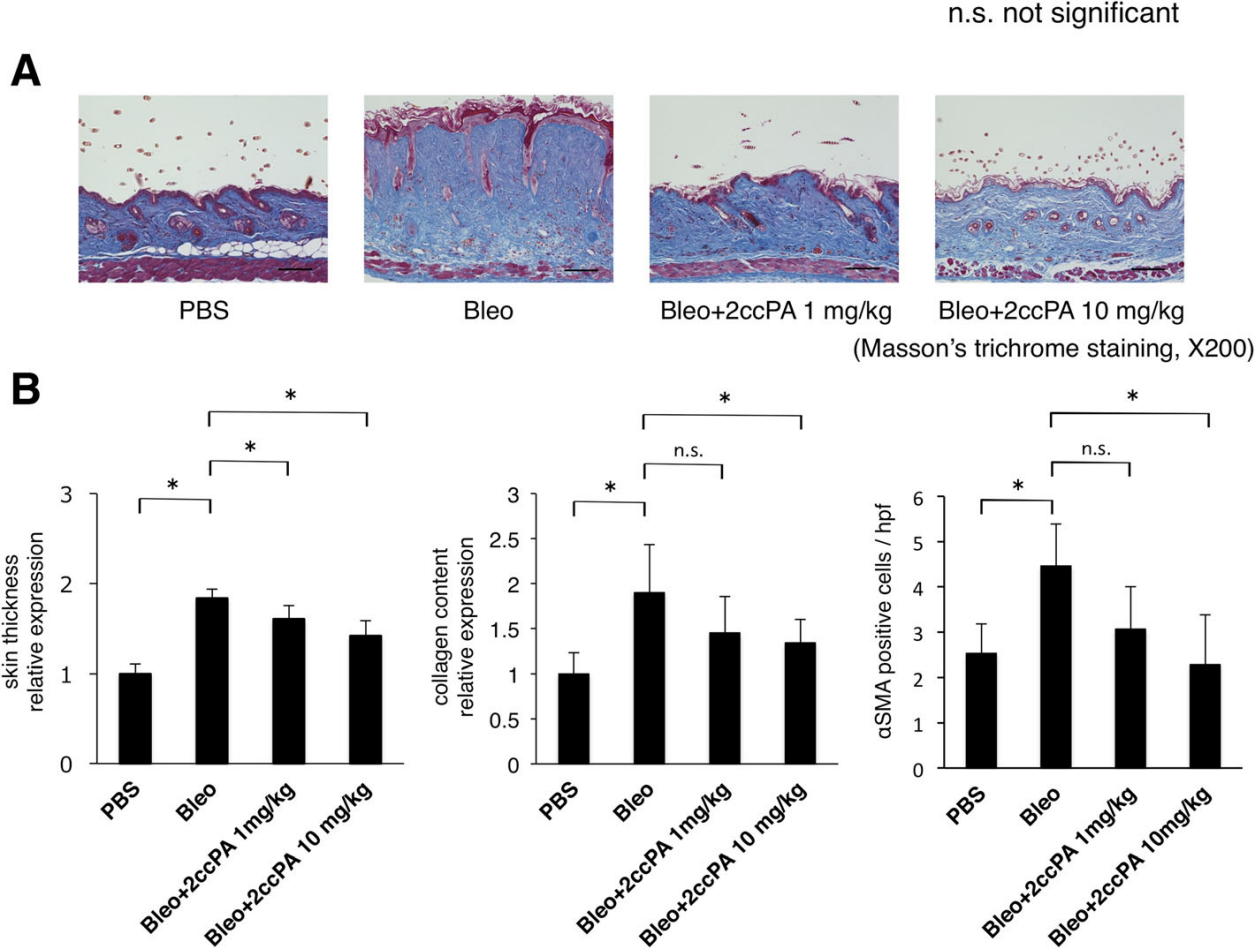
2ccPA suppressed the development of bleomycin-induced skin fibrosis in mice. **a** Representative histological sections in skin biopsies of mice treated with PBS, bleomycin (Bleo), Bleo+2ccPA 1 mg/kg, or Bleo+2ccPA 10 mg/kg. Scale bar = 100 μm. **b** Skin thickness, collagen content, and αSMA-positive cells of each group. Each group consisted of eight mice. Bars represent the mean ± SD. *p < 0.05

To further investigate the antifibrotic effect of 2ccPA, we used a mouse model of bleomycin-induced skin fibrosis. Mice were injected with PBS or 300 μg of bleomycin subcutaneously on their back 5 days per week for 4 weeks, and in the 2ccPA-treated group, mice were intraperitoneally injected with 2ccPA concurrent with a bleomycin inoculation. In 2ccPA-treated mice, no side effects were identified, and their body weights were not significantly reduced compared with bleomycin-treated mice. The skin thickness in the areas of bleomycin-induced skin fibrosis was significantly lower in the 2ccPA-treated group than in the no treatment group (Fig. 6b). Similarly, the collagen contents were reduced by 22.5% in the 1 mg/kg/day 2ccPA treatment group (not significant) and 32% in the 10 mg/kg/day 2ccPA treatment group (*p* < 0.05) compared with bleomycin-induced skin fibrosis (Fig. 6a, b). Similarly, the myofibroblast count, assessed by αSMA immunostaining, was reduced by 45% in the 10 mg/kg/day 2ccPA treatment group (*p* < 0.05) (Fig. 6b). Collectively, 2ccPA also exerted antifibrogenic effects in vivo*.*

## Discussion

Herein, we demonstrated the antifibrotic effect of 2ccPA on skin fibrosis. 2ccPA decreased the expression levels of LPA-driven *CTGF* and *IL-6* mRNAs in skin fibroblasts. Furthermore, we found that 2ccPA had direct effects on SSc skin fibroblasts to diminish the production of ECM molecules and on normal skin fibroblasts stimulated with TGF-β1. 2ccPA also elevated the levels of the antifibrogenic factors HGF and PGE_2_ in the culture medium of SSc skin fibroblasts. We further showed the preventive effect of 2ccPA on the development of bleomycin-induced skin fibrosis in mice.

Although the physiological activity of 2ccPA was not fully elucidated, inhibition of the growth and metastasis of cancer cells, survival and neurogenic pain and promotion of nerve cell differentiation, pain relief, and hyaluronic acid synthesis have been reported and expected for clinical use [18]. These properties are supported by the intrinsic biological properties of cPA, including antimitogenic regulation of the cell cycle, stress fiber formation, inhibition of platelet aggregation, Cl^−^ current activation, neuron overgrowth and survival enhancement, and mobilization of intracellular calcium [17, 18, 33]. In particular, we focused on intracellular cAMP elevation because intracellular cAMP levels have been implicated to regulate the fibrogenic status and intracellular cAMP-elevating agents can ameliorate fibrosis. The reported antifibrotic actions of cAMP are as follows: inhibition of the formation and action of profibrotic growth factors, inhibition of fibroblast formation, inhibition of myofibroblast conversion, initiation of fibroblast cell death, and inhibition of ECM production [34–36]. It is noteworthy that cyclosporine may improve focal segmental glomerulosclerosis by increasing intracellular cAMP levels in addition to its direct inhibitory effects on cells such as T cells and podocytes [37, 38]. According to our hypothesis, 2ccPA treatment increased the intracellular cAMP levels in skin fibroblasts. Intracellular cAMP levels are mainly elevated by AC stimulation and decreased by PDE [39]. 2ccPA binds to the LPA receptor. The LPA_4_ receptor is unique to other LPA receptors and only shares 20–24% amino acid sequence identity with LPA_1_ [17, 40–42]. Stimulation of LPA_4_ couples the Gs subunit and thus activates AC, whereas LPA_1_ and LPA_3_ signaling inhibits AC activation [42]. It seems strange that cPA has an antagonistic property towards LPA regardless of binding to the same LPA receptor; one explanation is the difference in receptor affinity: LPA has a high affinity for LPA_1–3_, while cPA has a high affinity for LPA_3–5_ [17]. These findings suggested that 2ccPA exerted antifibrotic effect via LPA_4_/AC/cAMP signaling, although we did not check the affinity of 2ccPA for LPA_4_ and that should be investigated for further research. Another possibility for the regulation of intracellular cAMP levels by 2ccPA is its action as a PDE3 inhibitor [33]. 2ccPA has been suggested to be an inhibitor of cPA-dependent decreased PDE3B expression in the HT-29 colon cell line [33]. The novel action of cPA as a PDE inhibitor should be elucidated in further investigations.

We also showed that 2ccPA promoted HGF and PGE_2_ production from SSc skin fibroblasts, which may be another antifibrotic mechanism of 2ccPA. HGF is known to be an epithelial repair molecule [30–32, 43]. HGF facilitates apoptosis of αSMA-positive myofibroblasts and enhances the expression of matrix metalloproteinases [43]. Overexpression of the HGF receptor c-met in SSc skin fibroblasts and high serum concentrations of HGF in SSc patients have been reported, suggesting a negative feedback response in the pathogenesis of SSc [44]. Altered PGE_2_ production and signaling have been noted in fibrotic fibroblasts. PGE_2_ has several inhibitory actions on fibroblast function, including chemotaxis, growth factor receptor expression, collagen synthesis, and myofibroblast differentiation [45]. PGE_2_ levels have been reported in bronchoalveolar lavage fluid in patients with idiopathic pulmonary fibrosis (IPF) [45]. In addition, fibroblasts derived from IPF patients have been shown to be decreased in EP_2_ (a PGE_2_ receptor) and protein kinase A (PKA) expression [46]. Thus, 2ccPA may regulate the physiological status of tissue fibrosis by decreasing ECM production and increasing antifibrotic molecules.

Elevated intracellular cAMP activates PKA, an exchange protein directly activated by cAMP (Epac) and the nucleotide-gated channel [35]. PKA is a cAMP-dependent protein kinase that phosphorylates serine/threonine residues of its target proteins. Once PKA is activated, the monomer catalytic subunit translocates to the nucleus and phosphorylates cAMP response element binding protein (CREB). CREB phosphorylation is also induced by mitogen-activated protein kinases (MAPKs) and Ca^2+^/ calmodulin-dependent protein kinases (CaMKs) [47]. Phosphorylated CREB binds to the coactivator CBP/P300 and promotes cAMP-response element (CRE)-dependent gene expression. In mammalian cells, CRE mediates various gene expression profiles related to cell survival, proliferation, and differentiation. The contribution of the cAMP/PKA pathway to the antifibrotic effect has been reported to be TGF-β/Smad-dependent [35]. CREB seems to inhibit interactions between Smad3 and CREB-binding protein (CBP) and p300, but not the nuclear translocation and DNA binding of the Smad3/4 complex [48]. Similarly, a cAMP-elevating agent did not abolish TGF-β1-driven Smad3 phosphorylation (data not shown). The other activated protein, Epac, has also been shown to play a role in the antifibrotic mechanisms of cAMP-elevating agents [49]. Epac is a PKA-independent guanine nucleotide exchange factor (GEF) that regulates the activities of G-proteins, such as Rap-1, by binding to GTP. The antifibrotic effect of Epac has been intensively investigated in the heart. Expression of Epac is regulated by profibrogenic stimuli (e.g., TGF-β and angiotensin II), and overexpression of Epac1 leads to decreased ECM production in cardiac fibroblasts [50]. Thus, it has been demonstrated that both PKA and Epac exert antifibrotic effects in a different manner. Due to the limitations of the currently available assays, it remains unclear which physiological factor determines the affinity of cAMP to PKA or Epac.

There are several limitations to our study. First, SSc is a heterogeneous disease, and a small number of skin fibroblasts do not represent all of the pathogenesis of the disease. Second, we did not perform loss and gain of function experiments. Therefore, we did not fully elucidate the relationship between LPA receptors and 2ccPA. Furthermore, we did not fully investigate how 22cPA increases the intracellular cAMP levels.

In summary, we demonstrate a novel property of 2ccPA in preventing the progression of fibrosis using SSc skin fibroblasts and bleomycin-induced skin fibrosis. 2ccPA abrogates ECM production and promotes antifibrotic molecules from SSc skin fibroblasts. 2ccPA also attenuates the progression of fibrosis in bleomycin-induced skin fibrosis. These findings suggest that 2ccPA may be a promising antifibrotic agent for the treatment of SSc.

## Conclusions

Antifibrotic treatment of SSc is still under development. Currently, severe skin fibrosis in SSc can be treated by immunosuppressive drugs. However, no clinical trial has proved that immunosuppressants are effective in treating SSc. 2ccPA, a stable derivative of cPA, is expected to be used in various clinical settings owing to its pleiotropic property. The ECM production of SSc skin fibroblasts was significantly reduced by 2ccPA in part via intracellular cAMP synthesis. Moreover, the antifibrotic effect was demonstrated using bleomycin-induced SSc model mice. It was suggested that 2ccPA could be a novel strategy for the treatment of SSc.

## Abbreviations

2ccPA: 2-Carba cyclic phosphatidic acid
ATX: Autotaxin
CTGF: Connective tissue growth factor
ECM: Extracellular matrix
GPCR: G protein-coupled receptors
LPA: Lysophosphatidic acid
LPC: Lysophosphatidylcholine
PDGF: Platelet-derived growth factor
SSc: Systemic sclerosis
TGF-β: Transforming growth factor-β
